# 2336 Novel PGF2a synthesis pathway in epithelial ovarian cancer

**DOI:** 10.1017/cts.2018.103

**Published:** 2018-11-21

**Authors:** Muhan Hu, Ekta Tiwary, Rebecca Arend, Michael Miller

**Affiliations:** University of Alabama at Birmingham

## Abstract

OBJECTIVES/SPECIFIC AIMS: To understand the role of PGF2a and to characterize a novel cyclooxyrgenase (COX)-independent prostaglandin synthesis pathway in epithelial ovarian cancer. METHODS/STUDY POPULATION: We used high grade epithelial ovarian cancer cell line (OVCAR3) as a model to study our pathway. Our main mode of PGF2a detection is through mass spectrometry. RESULTS/ANTICIPATED RESULTS: Our current results suggest the OVCAR3 cells may synthesize PGF2a independently of COX enzymes. We anticipate this novel pathway may be dependent on the TGFb pathway. DISCUSSION/SIGNIFICANCE OF IMPACT: Understanding the role and synthesis pathway of PGF2a may allow us to uncover a novel therapeutic pathway for high grade ovarian cancer.

